# Editorial Comment on Antibiotic Prophylaxis in Men Undergoing Transurethral Resection of Prostate: A Systematic Review and Network Meta‐Analysis

**DOI:** 10.1111/iju.70295

**Published:** 2025-11-24

**Authors:** Takanori Sekito, Hidetada Hirakawa, Takuya Sadahira

**Affiliations:** ^1^ Department of Urology Okayama University Graduate School of Medicine, Dentistry and Pharmaceutical Sciences Japan; ^2^ Department of Bacteriology Gunma University Graduate School of Medicine Japan

Transurethral resection of the prostate remains a cornerstone surgical therapy for benign prostatic hyperplasia; however, postoperative urinary tract infection (UTI) and urosepsis continue to pose challenges. The systematic review and network meta‐analysis by Oliveira et al. address the important clinical issue of determining optimal antimicrobial prophylaxis regimens to reduce postoperative infectious complications in this population [1]. By synthesizing randomized trials and indirect comparisons among multiple antimicrobial classes, their analysis demonstrates that prophylaxis markedly lowers postoperative bacteriuria, septicemia/bacteremia, fever, and length of stay compared with no antimicrobial use, with varying protective effects across antimicrobial classes depending on the outcome measured. Importantly, extending prophylaxis (> 72 h) did not provide consistent additional benefit and was associated with a nonsignificant trend toward higher septicemia/bacteremia risk, highlighting the importance of avoiding unnecessary antimicrobial exposure. This comprehensive work advances previous pairwise meta‐analyses by ranking regimens through network methodology and clarifying that short perioperative regimens targeting common uropathogens achieve the best balance between efficacy and safety.

Beyond reinforcing the value of prophylaxis, this study highlights several stewardship‐relevant insights. More intensive or prolonged regimens did not further reduce infection risk, supporting guideline‐based minimalist approaches. Recent best practice guidelines recommend single‐dose prophylaxis for most urologic procedures, with no additional postoperative dosing, citing no added benefit from extended regimens and a potential for increased antimicrobial resistance [2]. Moreover, the recent multicenter randomized trial suggested that, despite shorter antimicrobial prophylaxis application, microbiologically documented postoperative UTI rates tended to be lower and showed little difference between single‐dose and multi‐day regimens. While the study did not reach its planned sample size and cannot provide a confirmatory answer regarding noninferiority, the findings support the plausibility that prophylaxis duration could safely be reduced to the necessary minimum in transurethral prostate surgery [3].

In Oliveira et al.'s meta‐analysis, variation in infection definitions and resistance reporting across the included trials limits the generalizability of the findings to modern practice, where multidrug‐resistant organisms are becoming increasingly prevalent. A further consideration involves preoperative asymptomatic bacteriuria (ASB), which was not addressed in the meta‐analysis but remains clinically relevant. Recent data in the holmium laser enucleation of prostate, another major transurethral surgery, suggest that untreated ASB does not increase postoperative UTI or urosepsis, suggesting doubts about the necessity of universal treatment before these transurethral procedures [4]. Current European Association of Urology guidelines recommend screening for and treating ASB before mucosa‐breaching urologic surgery, and emphasize tailoring therapy to individual risk and local resistance data [5]. While Oliveira et al. provide a valuable ranking of prophylactic strategies, clinicians should interpret these results within the framework of antimicrobial stewardship—selecting agents based on institutional antibiograms, limiting duration to a single preoperative dose, and avoiding unnecessary broad‐spectrum exposure. Ongoing stewardship efforts and well‐designed prospective trials will be essential to refine prophylactic policies, ensuring infection prevention without accelerating antimicrobial resistance in common transurethral prostate surgeries.

## Author Contributions

Takanori Sekito: conceptualization, writing – original draft, Hidetada Hirakawa: writing – review and editing, Takuya Sadahira: writing – review and editing.

## Conflicts of Interest

The authors declare no conflicts of interest.

## Linked Article

Alessandro V. Oliveira et al. Antibiotic Prophylaxis in Men Undergoing Transurethral Resection of Prostate: A Systematic Review and Network Meta‐Analysis. Doi: https://doi.org/10.1111/iju.70276.
